# Beyond Temperature: Relative Humidity Systematically Shifts Juvenile Thermal Performance and Projected Population Growth in a Malaria Vector

**DOI:** 10.1111/ele.70416

**Published:** 2026-06-11

**Authors:** Paul J. Huxley, Joel J. Brown, Brandyce St. Laurent, Britny Johnson, Olivia Y. Cheung, Anna Asamoah, Brandon D. Hollingsworth, Eric R. Bump, Michael C. Wimberly, Mercedes Pascual, Leah R. Johnson, Courtney C. Murdock

**Affiliations:** ^1^ Department of Biology University of York UK; ^2^ Department of Infectious Disease Epidemiology Imperial College London London UK; ^3^ Department of Statistics Virginia Tech Blacksburg Virginia USA; ^4^ Department of Entomology Cornell University Ithaca New York USA; ^5^ Cornell Institute for Host Microbe Interaction and Disease Cornell University Ithaca New York USA; ^6^ Department of Epidemiology and Biostatistics, Arnold School of Public Health University of South Carolina Columbia South Carolina USA; ^7^ Department of Geography and Environmental Sustainability University of Oklahoma Norman Oklahoma USA; ^8^ Data Institute for Societal Challenges University of Oklahoma Norman Oklahoma USA; ^9^ Department of Biology and Environmental Studies New York University New York USA

**Keywords:** *Anopheles stephensi*, climate suitability, epidemiology, humidity, malaria, mosquito, population growth rate, temperature dependence, thermal performance curve, vector‐borne diseases

## Abstract

Understanding how temperature‐sensitive organisms respond to environmental change is central to addressing challenges in public health, biodiversity conservation and food security. For many ectotherms, abiotic and biotic factors shape their abundance and distribution by generating stage‐specific variation in life history traits. Although previous studies have examined temperature, rainfall, competition and habitat quality in relation to maximal population growth rate ($r_{m}$), relative humidity has rarely been incorporated into trait‐based thermal performance frameworks. Using laboratory experiments, we show that relative humidity alters juvenile life history trait responses in *Anopheles stephensi*, an important malaria vector. We then integrate these humidity‐dependent juvenile trait responses into an analytic $r_{m}$ model to examine how relative humidity shifts the temperature dependence of projected population growth. Heuristic climate‐suitability comparisons further illustrate that temperature–humidity interactions acting through juvenile traits alone can alter qualitative inference about when and where temperature‐only models may over‐ or underestimate environmental suitability. These results highlight the importance of incorporating humidity alongside temperature when assessing ectotherm responses to climatic variation.

## Graphical abstract

Understanding how ectotherms respond to environmental change requires accounting for multiple interacting climatic factors; yet relative humidity is rarely integrated with temperature in trait‐based performance frameworks. Using laboratory experiments and an analytic population growth model, we show that humidity systematically modifies juvenile life history traits and shifts the temperature dependence of projected maximal population growth in *Anopheles stephensi*, an important malaria vector. Illustrative climate comparisons show that incorporating humidity can qualitatively alter inferences about environmental suitability relative to temperature‐only approaches, highlighting the importance of integrating additional environmental drivers into mechanistic models of ectotherm performance.

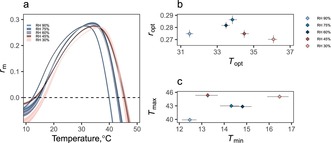

## 1 Introduction

Arthropods, including mosquitoes, experience a complex suite of environmental drivers, including abiotic factors such as temperature, rainfall, humidity and salinity, as well as biotic factors such as natural enemies, inter‐ and intraspecific competition, and variation in habitat quality. Yet, in vector ecology—and more broadly in ecology—there has been a strong emphasis on temperature as the primary determinant of changes in organismal abundance and distribution under climate change (reviewed in Mordecai et al. 2019). Temperature effects on ectotherm performance are typically non‐linear, with biological rates increasing from zero at a minimum temperature ($T_{\min}$), peaking at an intermediate optimum ($T_{\mathrm{pk}}$) and then declining sharply towards a critical upper temperature where mortality occurs ($T_{\max}$). These responses arise because body temperature directly influences enzymatic reaction rates and the structural integrity of cellular membranes and proteins (Angilletta 2009). Together, these features give rise to thermal performance curves (TPCs), which are widely used to infer ecological and evolutionary responses to contemporary and future temperature variation (Huey and Kingsolver 2019; Pawar et al. 2024; Mordecai et al. 2019; Miazgowicz et al. 2020; Tesla et al. 2018).

In addition to temperature, water availability is a fundamental abiotic constraint on ectotherm biology, and both factors jointly influence the abundance and distribution of organisms (reviewed in Brown et al. 2023; Rozen‐Rechels et al. 2019). For mosquitoes that transmit human pathogens, water availability plays a particularly important role because juvenile stages develop in aquatic habitats that directly constrain population growth. Due to the thermodynamic relationship between temperature and the amount of moisture air can hold (Brown et al. 2023), variation in relative humidity and temperature can alter evaporation rates in larval environments (Juliano and Stoffregen 1994) as well as intrinsic properties of these habitats, including solute concentration (Juliano and Stoffregen 1994) and surface tension (Pérez‐Díaz et al. 2012; Singh and Micks 1957). Through these pathways, relative humidity has the potential to reshape juvenile thermal environments even under constant water input.

Despite extensive work on temperature‐dependent performance and on humidity or desiccation limits in terrestrial life stages, explicit tests of temperature × relative humidity interactions in insects with aquatic juvenile stages remain uncommon. This gap is notable because evaporation dynamics and air–water interface processes can decouple ambient air temperature from the conditions experienced by developing juveniles, allowing humidity to modify thermal responses in non‐additive ways. Although a small number of laboratory studies have examined combined effects of temperature and relative humidity on insect performance (e.g., Gol'berg 1982; Yadav and Chaudhary 1986; Rocha et al. 2001; Simelane 2007; Chiarelli et al. 2011; Kumar et al. 2013), these studies have focused almost exclusively on terrestrial life stages or adult traits and have not embedded humidity effects within trait‐based thermal performance or population growth frameworks. Consequently, how temperature–humidity interactions structure juvenile traits and downstream population fitness in insects with aquatic life stages remains largely unexplored.

Recent syntheses argue more broadly that temperature‐only thermal performance approaches can misrepresent organismal vulnerability because thermal performance curves themselves depend on other environmental drivers, including resource availability, water balance and additional abiotic and biotic stressors (Sinclair et al. 2016, 2024; Litchman and Thomas 2023). Across diverse taxa, stressful environmental conditions often lower thermal optima and upper thermal limits, increasing susceptibility to warming (Litchman and Thomas 2023). These frameworks explicitly call for empirical studies that incorporate humidity and other non‐thermal drivers into trait‐based thermal performance analyses, rather than treating them only as secondary or correlative modifiers. Here, we investigate how variation in relative humidity during juvenile development alters the temperature dependence of juvenile traits and projected maximal population growth rate ($r_{m}$) in *Anopheles stephensi*, a South Asian urban malaria vector that has recently expanded in Africa (Zhou et al. 2024). We conducted a large laboratory experiment in which *An. stephensi* larvae were exposed to eight core constant temperatures (16°C–40°C), additional lower and upper limit treatments (14°C and 42°C), five constant relative humidity levels (30%–90%) and two contrasting evaporation scenarios. Specifically, we quantified how relative humidity modifies the temperature dependence of key juvenile traits—development rate, survival probability and adult body size at emergence—and then integrated the juvenile responses into an analytic $r_{m}$ framework to examine how humidity shifts the thermal limits and optimum of projected population growth. By explicitly incorporating humidity into a juvenile trait‐based thermal framework, our study tests whether temperature‐only approaches can qualitatively bias inference about environmental suitability.

## 2 Materials and Methods

### 2.1 Experimental Set–Up

Briefly, we reared *An. stephensi* larvae across constant temperatures (14°C–42°C; core range 16°C–40°C), five relative humidity levels (30%–90%) and two evaporation regimes (water volume controlled vs. allowed to evaporate; see Supplementary Methods for detailed description). Each treatment included three replicate trays containing 100 first‐instar larvae under a 12:12 light:dark cycle (Table S1). The experimental unit was the tray: larvae within a tray shared water volume, food inputs, density and microenvironment and were therefore treated as subsamples rather than independent replicates. Treatments were replicated across temporally independent blocks (distinct cohorts) in which the full temperature × relative humidity design was run concurrently, thereby preventing confounding with time or incubator identity. Relative humidity and temperature gradients were chosen to span conditions across the species' native and invasive range.

### 2.2 Measuring Life History Trait Performance

We recorded daily emergence to estimate juvenile survival (proportion emerging) and development time. Adult female wing length was measured as a proxy for body size (*n* = 20 females per tray per treatment; see Supplementary Methods for detailed description).

### 2.3 Modelling Thermal Performance of Juvenile Traits Across Relative Humidity Gradients

We quantified humidity‐specific thermal performance curves for juvenile development rate and survival using the bayesTPC package in R (Sorek et al. 2025; R Core Team 2023). Development rate and survival TPCs were fitted separately for each humidity level, and thermal limits and optima ($T_{\min}$, $T_{\mathrm{pk}}$, $T_{\max}$) were extracted from posterior distributions (Supporting Information Equations S1–S3). To test whether humidity modifies the temperature–size relationship, we fit hierarchical regressions of female wing length with temperature, relative humidity and their interaction as predictors, including tray as a random effect. Additional models tested whether development time predicted adult size after accounting for temperature and humidity (Supplementary Methods).

### 2.4 Modelling Temperature‐Dependent Mosquito Population Growth Across Relative Humidity Gradients

To examine the mechanistic implications of humidity‐driven changes in juvenile traits for population fitness, we integrated our temperature‐dependent juvenile trait relationships into an existing analytic model of mosquito maximal population growth rate ($r_{m}$). We used a continuous‐time, stage‐structured approximation based on the Euler–Lotka equation (Charnov 1993; Savage et al. 2004; Amarasekare and Savage 2012; Cator et al. 2020). In this context, the model is used as a trait‐based projection framework to isolate how humidity‐induced changes in juvenile traits could shift the temperature dependence of $r_{m}$, rather than as a full demographic model of realized mosquito population dynamics. Cator et al. (2020) derived an approximation appropriate for the range of growth rates typically seen across arthropods (Equation 1; Table 1):

(1) $$r_{m}\approx \frac{\left(\kappa +z\right)\left(\ln\left(\frac{b_{\max}}{\kappa +z}\right)-\alpha z_{J}\right)}{\alpha \left(\kappa +z\right)+1}.$$

**TABLE 1:** Definitions of model parameters.

Parameter	Units	Description
$r_{m}$	day^−1^	Maximal population growth rate
α	days	Juvenile‐to‐adult development time
$b_{\max}$	eggs × female^−1^ day^−1^	Maximum fecundity rate
κ	day^−1^	Fecundity loss rate
z	day^−1^	Adult mortality rate
$p_{\mathrm{EA}}$	probability	Survival averaged across juvenile stages
$T_{\mathrm{pk}}$	°C or K	Temperature at which trait performance peaks
$T_{\min}$	°C or K	Lower temperature at which trait performance ceases
$T_{\max}$	°C or K	Upper temperature at which trait performance ceases
$B_{\mathrm{pk}}$	Measurement unit of trait	Trait performance achieved at $T_{\mathrm{pk}}$
$T_{\mathrm{opt}}$	°C	Temperature at which $r_{m}$ peaks
$r_{\mathrm{opt}}$	day^−1^	$r_{m}$ achieved at $T_{\mathrm{opt}}$

Here, α is the juvenile‐to‐adult development time (days), $b_{\max}$ is the peak reproductive rate (individuals (eggs) × individuals (females) × day^−1^), κ is the fecundity loss schedule (individual^−1^ day^−1^), and $z_{J}$ and z are juvenile and adult mortality rates (individual^−1^ day^−1^), respectively.

In this study, our goal is to isolate how relative humidity modifies the temperature dependence of juvenile traits and, through them, projected $r_{m}$. We therefore held adult mortality and fecundity (z and $b_{\max}$ in Equations 1 and 2) constant across temperature × humidity treatments at 0.12 day^−1^ and 11.37 day^−1^, respectively. These values were estimated as trait means from a literature synthesis for *An. stephensi* (Supporting Information file). Holding selected parameters constant to isolate the contribution of others is standard practice across experimental science, mechanistic modelling and sensitivity analysis. Here, it allows the contribution of humidity‐driven juvenile trait variation to be evaluated directly, without introducing additional sources of variation from adult traits that were not measured in the present experiment. Because Equation (1) is an approximation, it is most accurate when $r_{m}<1$ day^−1^ (Cator et al. 2020), which is typically the case for insects (Frazier et al. 2006; Pawar et al. 2024). Further details of the derivation and assumptions are given in Cator et al. (2020).

Juvenile mortality rate ($z_{J}$ in Equation 1) is not directly observable from the data collected in this study. Instead, we measure the probability of emergence as an adult (also referred to as juvenile‐to‐adult survival probability), which we denote as $p_{\mathrm{EA}}$. Under the assumption of a constant juvenile mortality rate, survival through the development period of duration α is given by

$$p_{\mathrm{EA}}=\mathrm{Pr}\left(\text{survival through development}\right)=e^{-\alpha z_{J}}$$

Taking the natural logarithm gives

$$\ln\left(p_{\mathrm{EA}}\right)=-\alpha z_{J}$$

Substituting $\ln\left(p_{\mathrm{EA}}\right)$ for $-\alpha z_{J}$ in Equation (1) yields the modified expression for maximal population growth rate:

(2) $$r_{m}\approx \frac{\left(\kappa +z\right)\left[\ln\left(\frac{b_{\max}}{\kappa +z}\right)+\ln\left(p_{\mathrm{EA}}\right)\right]}{\alpha \left(\kappa +z\right)+1}.$$

As κ has been shown to only make a very small contribution to $r_{m}$ (Cator et al. 2020), we assume that $b_{\max}$ declines with age at a constant rate of 0.01 individual^−1^ day^−1^. Finally, to obtain all quantities of interest for $r_{m}$ ($r_{\mathrm{opt}}$, $T_{\mathrm{opt}}$, $T_{\min}$ and $T_{\max}$), we used a similar procedure to that used for survival. However, for $r_{m}$ $T_{\min}$ and $T_{\max}$, we used the TPC posteriors at each humidity level to estimate the temperatures at which $r_{m}$ was zero. Note that we refer to the temperature of peak $r_{m}$ as $T_{\mathrm{opt}}$ (Table 1) rather than $T_{\mathrm{pk}}$ as we do for traits because the $T_{\mathrm{pk}}$s of those traits do not necessarily correspond to the thermal optimum of population fitness (optimal thermal fitness; Pawar et al. 2024).

We decomposed the temperature sensitivity of $r_{m}$ into trait‐specific contributions using the chain rule (Supporting Information Equation S4). To isolate humidity effects on juvenile traits, adult mortality and fecundity were held constant; robustness to alternative adult parameter values is shown in the Supporting Information (Figure S3).

### 2.5 Mapping Climatic Suitability for Mosquito Population Growth

We used historical gridded climate data (NASA NEX‐GDDP‐CMIP6, 1970–2000) to compare seasonal $r_{m}$ predicted by a temperature‐only model versus a temperature × humidity model across Africa and South Asia. These spatial analyses are intended as heuristic comparisons of model structure—illustrating how inclusion of humidity‐dependent juvenile traits changes qualitative inference relative to a temperature‐only framework—rather than as predictive maps of realized abundance, establishment or distribution. Methods and climate processing are detailed in the Supporting Information.

## 3 Results

### 3.1 Effects of Relative Humidity on the Thermal Performance of Juvenile Traits

**FIGURE 1:**
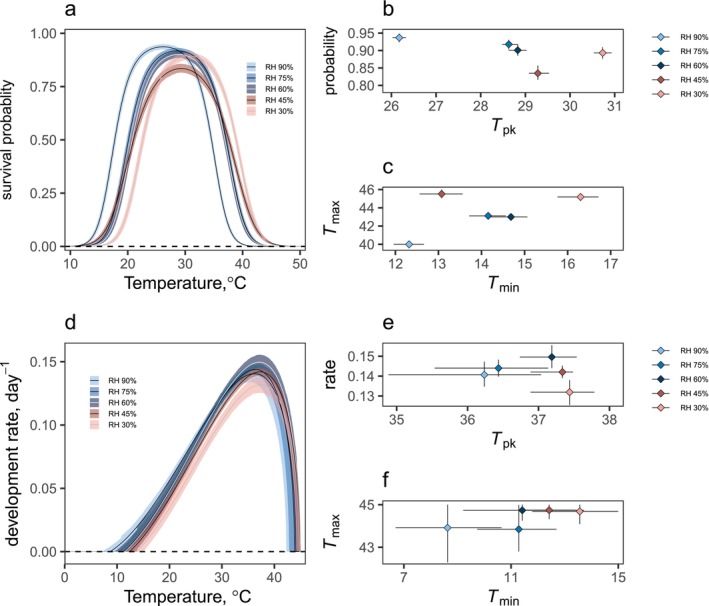
Relative humidity shapes the temperature dependence of juvenile fitness traits in *Anopheles stephensi*. The effects of relative humidity variation on the thermal performance curves (TPCs) of juvenile survival probability ($p_{\mathrm{EA}}$ in Equation 2), and juvenile development rate (1/α in Equation 2). Shading around the lines are HPD intervals calculated using the posteriors for each humidity‐dependent TPC. Point‐range summaries represent how the predicted thermal peaks ($T_{\mathrm{pk}}$), and minimum ($T_{\min}$) and maximum ($T_{\max}$) vary at each relative humidity level for each trait. Bidirectional error bars are 95% CrIs. Survival probability, $p_{\mathrm{EA}}$: (a) posterior distribution of fitted TPCs across humidity levels; (b) maximum survival probability vs. $T_{\mathrm{pk}}$; (c) $T_{\min}$ and $T_{\max}$. Development rate, 1/α: (d) posterior distribution of fitted TPCs across humidity levels; (e) maximum development rate vs. $T_{\mathrm{pk}}$; (f) $T_{\min}$ vs. $T_{\max}$. The observed development‐time data used to derive the development‐rate curves are shown in Figure S2. In all panels, bidirectional error bars are 95% credible intervals. Differences among humidity treatments are summarized by shifts in posterior medians and associated credible intervals, which quantify uncertainty in thermal limits and optima.

Posterior estimates indicate that relative humidity systematically shifts thermal performance of juvenile traits, with posterior distributions showing clear shifts in TPC parameters across humidity levels (Figure 1a–c; Table S1). While variation in relative humidity resulted in changes to the predicted $T_{\min}$, $T_{\mathrm{pk}}$ and $T_{\max}$, the overall shape of the curve remained qualitatively similar for both the probability of juvenile survival (symmetric, quadratic function, Figure 1a) and juvenile development rate (asymmetric, Briere function, Figure 1d).

Across both traits, decreases in relative humidity consistently shifted thermal performance toward warmer temperatures, indicating that humidity shifted the location of juvenile thermal performance curves while preserving their overall form. Lower relative humidity shifted juvenile thermal limits and optima toward warmer temperatures by approximately 1°C–5°C depending on trait (Figure 1; Tables S2–S5). Additionally, at temperatures that optimized the probability of juvenile survival ($T_{\mathrm{pk}}$), survival was ∼11% lower at low relative humidity (45% RH) than high relative humidity (90% RH) (Figure 1c, Table S3). In contrast for juvenile development rate, mosquitoes developed fastest (0.15 day^−1^ or ∼6.7 days) at intermediate humidity (60% RH) and lowest at extreme relative humidity (30% RH, 0.132 day^−1^ or ∼7.6 days; 90% RH, 0.141 day^−1^ or ∼7.1 days) at $T_{\mathrm{pk}}$ (Figure 1e, Table S4), indicating a non‐monotonic humidity response.

When evaporation was allowed to occur, mosquitoes developing in trays held at 30% or 45% RH did not survive to adulthood at any temperature, because trays in these treatments evaporated completely prior to adult emergence. Mortality under these conditions therefore reflects complete habitat loss due to desiccation rather than direct physiological constraints imposed by temperature or humidity per se. In general, we observed similar qualitative responses of juvenile traits to relative humidity variation, with decreases in relative humidity shifting thermal performance toward warmer temperatures (Figure S4, Tables S8–S11). However, the magnitude of these shifts was substantially amplified under evaporation, indicating that humidity effects on juvenile performance are strongest when evaporation alters the physical properties of the aquatic environment. Allowing for evaporation did not change the overall shape of the thermal performance curve for a given trait (e.g., survival probability = quadratic, Figure S4a; development rate = Briere, Figure S4d).

For survival probability, humidity effects occurred over a narrower RH range but with greater magnitude. For example, relative to minimal evaporation, the probability of juvenile survival declined much more steeply at intermediate RH when evaporation occurred, with survival at $T_{\mathrm{pk}}$ dropping by ∼35% at 60% RH compared to ∼3.8% under controlled evaporation. Notably, under evaporation the upper thermal limit ($T_{\max}$) remained constrained near ∼40°C across humidity levels, resulting in a pronounced narrowing of thermal breadth as humidity declined. For juvenile development rate, it was only possible to fit TPCs at the highest RH levels (75% and 90% RH) when evaporation occurred due to insufficient prior information at lower RH. In general, development TPCs were more thermally constrained under evaporation, with warmer $T_{\min}$ values and lower $T_{\mathrm{pk}}$ and $T_{\max}$ relative to minimal evaporation conditions (Figure S4d, Tables S11–S12).

### 3.2 Effects of Relative Humidity on Temperature–Size Relationships

**FIGURE 2:**
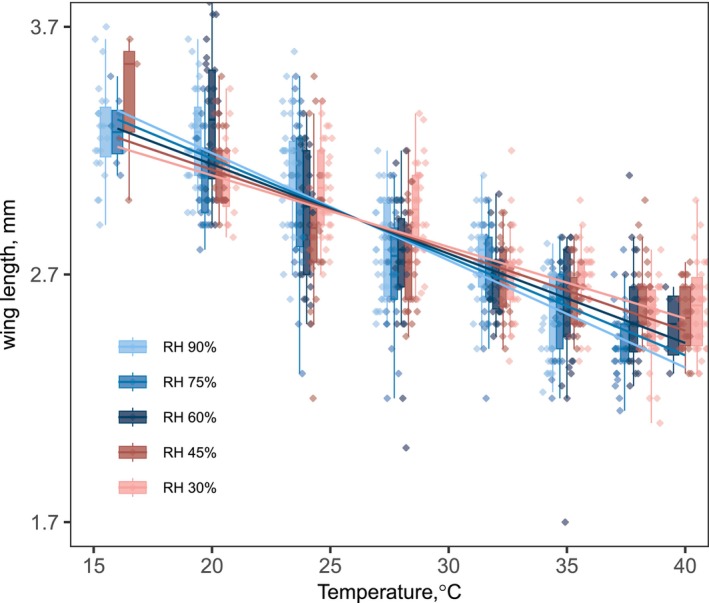
Relative humidity moderates the temperature–size relationship in *Anopheles stephensi*. Regression lines show the decline in wing size with temperature and how that decline is steeper at higher RH. Boxplot elements: Median (line), 25th/75th percentiles (hinges), whiskers extend to 1.5 × IQR; points = individual mosquitoes.

We found clear effects of temperature, relative humidity (RH) and their interaction on adult size at emergence (wing length; Figure 2). Wing length declined with increasing temperature (slope: −0.0216; 95% CrI: −0.0276,−0.0158), and this temperature–size decline became steeper at higher RH (Temp × RH: −0.0002; 95% CrI: −0.0003,−0.0001). Across 16°C–40°C, predicted size declined by ∼1 mm at 90% RH but by only ∼0.07 mm at 30% RH. Under uncontrolled evaporation, mean adult size was larger overall, but the interaction reversed: temperature‐driven declines in size were steepest at lower RH (Temp × RH: 0.00076; 95% CrI: 0.00042,0.00110; Figure S6). In these evaporative conditions, predicted size declined by ∼1.43 mm at 60% RH compared with ∼0.92 mm at 90% RH across the same temperature range. Fixed‐effect estimates were robust to alternative hierarchical structures (see Supporting Information).

After accounting for temperature, development time showed a weak but RH‐dependent association with adult size: near zero at low RH and increasingly positive at higher RH (Supporting Information). Together, these results indicate that relative humidity modifies both the magnitude and direction of temperature–size relationships, with effects contingent on evaporative conditions.

### 3.3 Effects of Relative Humidity on the Thermal Performance of Mosquito Population Growth Rate

**FIGURE 3:**
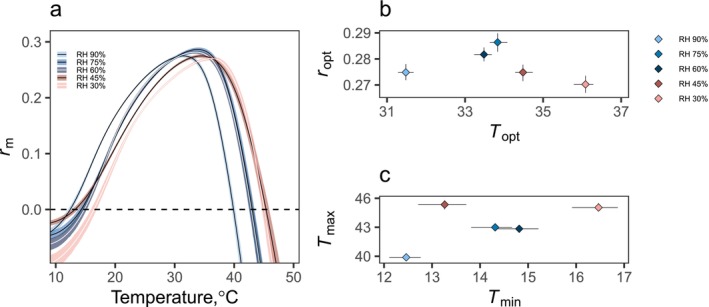
Effects of relative humidity on juvenile fitness traits shape the temperature dependence of maximal population growth rate, $r_{m}$. (a–c) a. $r_{m}$ TPCs across relative humidity levels. (b,c) b. $r_{\mathrm{opt}}$s versus $T_{\mathrm{opt}}$s, **c**. $T_{\min}$ versus $T_{\max}$ across humidity levels. Prediction bounds in a are HPD intervals calculated using the posteriors for each humidity–dependent TPC. Points (medians) in b and c were estimated numerically from the posterior distributions for each humidity level; bidirectional error bars are 95% credible intervals and summarize posterior uncertainty in $r_{\mathrm{opt}}$, $T_{\mathrm{opt}}$, $T_{\min}$ and $T_{\max}$ across humidity levels.

Across all humidity levels, maximal population growth rate ($r_{m}$) exhibited a unimodal, asymmetric relationship with temperature, remaining positive above ∼15°C and below ∼40°C, with optima between 0.27 and 0.29 day^−1^ (Figure 3a,b; Tables S6 and S7). As with juvenile life history traits, variation in relative humidity systematically shifted the thermal performance of $r_{m}$ (Figure 3a; Tables S6–S7). Decreases in relative humidity from 90% to 30% RH increased predicted $T_{\mathrm{opt}}$ by ∼4.6°C, $T_{\min}$ by ∼4°C and $T_{\max}$ by ∼5.1°C (Figure 3b,c; Table S7). At temperatures optimizing $r_{m}$, growth rates were highest at intermediate humidity levels (60%–75% RH; Table S6), mirroring the non–monotonic humidity responses observed in juvenile traits and indicating that population‐level outcomes primarily reflect humidity effects on early life stages.

**FIGURE 4:**
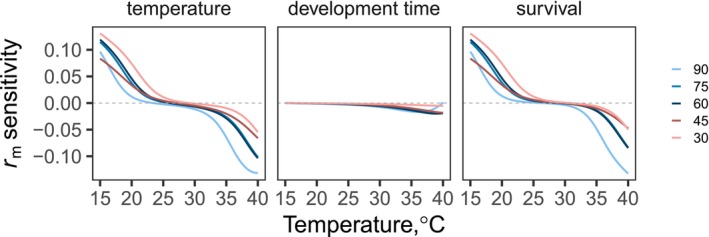
Sensitivity of maximal population growth rate, $r_{m}$ to juvenile trait responses. Estimates of $r_{m}$ are highly sensitive to temperature, driven almost entirely by sensitivity to juvenile survival ($p_{\mathrm{EA}}$ in Equation 2, Table 1). This sensitivity is highest at the low and high temperature extremes, with relatively low sensitivity from 22°C to 30°C. Relative humidity modulates the sensitivity of $r_{m}$ to $p_{\mathrm{EA}}$, by increasing sensitivity at warmer temperatures and decreasing sensitivity at cooler temperatures. $r_{m}$ shows relatively low sensitivity to juvenile development time (α in Equation 2, Table 1).

Under evaporation, humidity produced similar directional shifts in $r_{m}$ thermal performance curves (Figure S5, Tables S12 and S13), with comparable changes in TPC parameters across a similar humidity range as habitats with minimal evaporation. Absolute growth rates, however, were slightly higher in evaporative environments (mean $r_{m}$ = 0.297) than under controlled evaporation (mean $r_{m}$ = 0.286), with the highest growth rates occurring at 75% RH at optimal temperatures. Sensitivity analyses revealed that the temperature dependence of $r_{m}$ was driven primarily by juvenile survival, with smaller contributions from juvenile development time (Figure 4). At cooler temperatures ($<25^{\circ }$C), higher humidity dampened the sensitivity of $r_{m}$ to temperature via effects on juvenile survival, whereas above ∼30°C, higher humidity increased this sensitivity. Although $r_{m}$ was relatively insensitive to development time across most temperatures, relative humidity modestly increased the negative contribution of development time at warm temperatures, strengthening the inverse relationship between development time and $r_{m}$. Similar qualitative patterns were observed under higher‐evaporation conditions, where sensitivity to humidity via juvenile survival was greater at both thermal extremes and the dampening effect at low temperatures was most pronounced.

As a robustness check that preserved the original modelling framework, we recomputed $r_{m}$ using the same posterior fits for juvenile traits while simultaneously scaling adult mortality (z) and fecundity ($b_{\max}$) constants by ±25% and ±50%. These perturbations produced only modest shifts in $T_{\min}$, $T_{\max}$, $T_{\mathrm{opt}}$ and $r_{\mathrm{opt}}$, and did not alter the qualitative humidity‐driven patterns described above (Figure S3).

### 3.4 Climatic Suitability for Mosquito Population Growth

**FIGURE 5:**
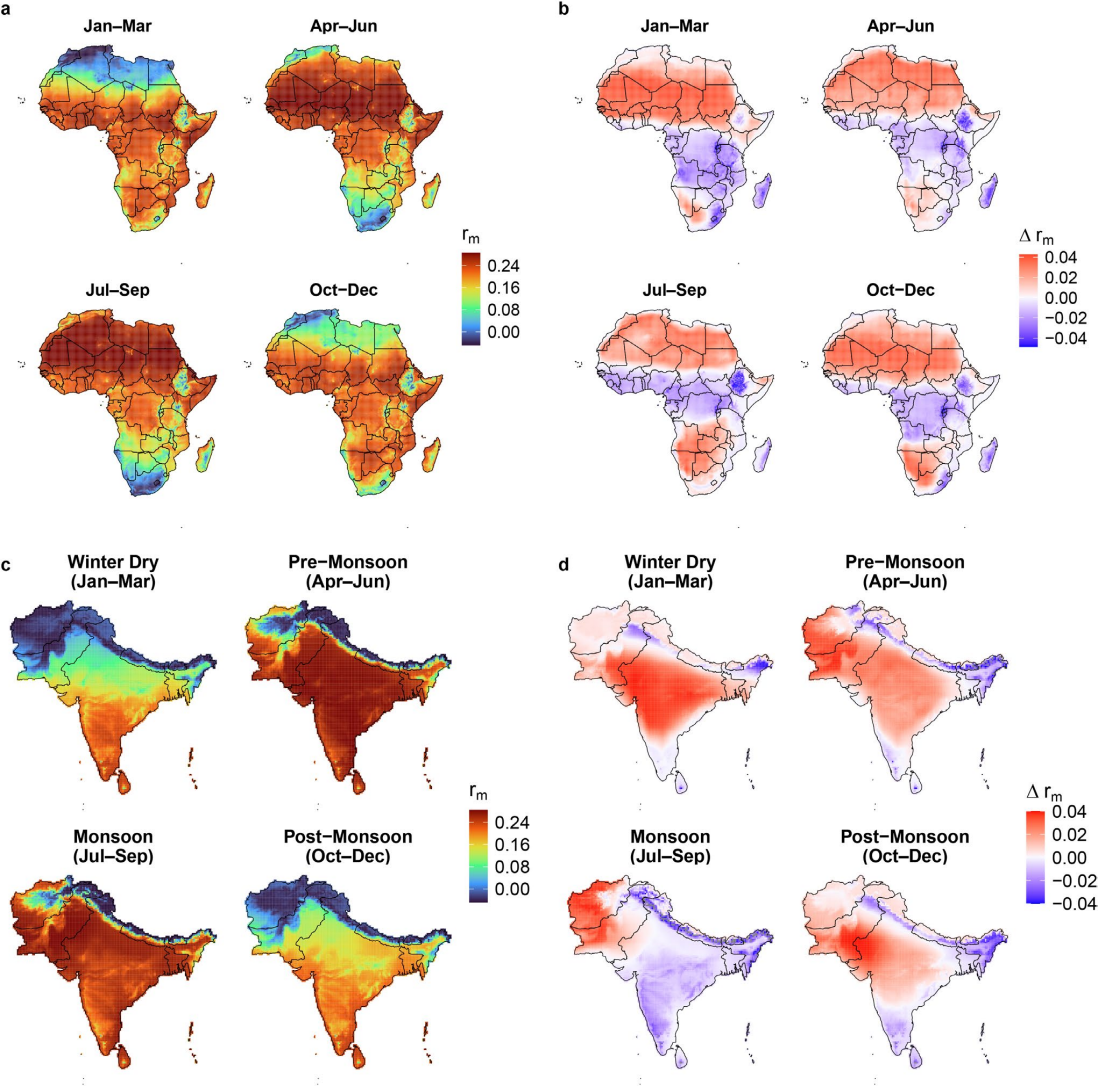
Seasonal spatial patterns of $r_{m}$ derived from the NASA NEX‐GDDP‐CMIP6 daily historical dataset (1970–2000), for Africa (a,b) and South Asia (c and d) based on temperature and humidity. Maps (a) and (c) show the seasonal differences in $r_{m}$ for the temperature‐only model. In maps (b) and (d), $\Delta r_{m}$ = temperature‐only $r_{m}$ minus temperature‐ and humidity‐dependent $r_{m}$. Red (positive) values indicate that the temperature‐only $r_{m}$ model overestimated $r_{m}$ and blue (negative) values indicate that temperature‐only $r_{m}$ model underestimated $r_{m}$ (i.e., the temperature and humidity model predicted higher $r_{m}$ than the temperature‐only model). White values denote that both models were in agreement ($\Delta r_{m}$ = 0). Note that these maps are heuristic, qualitative illustrations intended to compare model behaviour with and without humidity effects on juvenile traits, not predictive distribution maps.

To illustrate the qualitative implications of humidity‐driven juvenile trait variation, we compared seasonal population growth rates predicted by a temperature‐only framework with those predicted by a temperature × humidity framework. The temperature‐only model predicted higher $r_{m}$ values in warmer tropical regions, and lower values in cooler high‐elevation areas like the Himalayas, Atlas Mountains and Ethiopian Highlands (Figure 5a,c). Including humidity reduced $r_{m}$ in some of Africa's hottest and driest regions—parts of the Sahara, Sahel, southwest Africa and the Horn of Africa—while increasing it in most of central, southern and portions of North Africa, with the largest rises at higher elevations. These patterns were consistent year‐round, but areas where humidity increased $r_{m}$ shifted northward from January to June and southward from July to December, following the intertropical convergence zone and seasonal humidity (Figure 5b). In South Asia, humidity generally reduced $r_{m}$ across central India, but increased it in northern Pakistan, Bangladesh, southern India and Sri Lanka (Figure 5d). During the humid monsoon, humidity led to increased $r_{m}$ across most of India, especially central regions. The temperature‐only model indicated larger areas of suitable habitat: about 0.4 million km^2^ more in South Asia (3.1 vs. 2.7 million km^2^) and 0.8 million km^2^ more in Africa (25 vs. 24.2 million km^2^). In both Africa and South Asia, models with humidity predicted higher climate suitability during rainy seasons, while temperature‐only models indicated higher suitability during drier periods.

## 4 Discussion

Our results show that reductions in relative humidity during the aquatic developmental stages of *An. stephensi* systematically shift the temperature dependence of juvenile traits and, through them, the temperature dependence of projected maximal population growth rate ($r_{m}$), primarily via effects on juvenile survival. Relative humidity also modified the temperature–body size relationship in adults. Together, these findings show that variation in relative humidity can substantially alter thermal performance in aquatic juvenile stages and thereby change trait‐based inference about mosquito population fitness under different environmental conditions.

More broadly, our findings are consistent with recent syntheses arguing that thermal performance curves remain a useful mechanistic framework, but that temperature rarely acts alone and that additional environmental drivers can reshape thermal responses in ecologically important ways (Sinclair et al. 2016; Litchman and Thomas 2023). In particular, stressful environmental conditions often lower thermal optima and upper thermal limits, implying that temperature‐only approaches may underestimate vulnerability to warming (Litchman and Thomas 2023). Water balance has likewise been identified as a major but under‐incorporated component of predictions of insect responses to climate change (Brown et al. 2023; Sinclair et al. 2024). By explicitly examining how relative humidity modifies temperature‐dependent juvenile traits, our study addresses this recognized gap and extends trait‐based thermal performance analysis to a system with aquatic juvenile stages.

The temperature effects we observed are consistent with previous research showing non‐linear, unimodal temperature–trait relationships in mosquitoes (Mordecai et al. 2019). However, our TPC predictions for juvenile traits under intermediate RH levels differed from predictions reported in temperature‐only studies on *An. stephensi* (Villena et al. 2022), likely reflecting their use of synthesized data to fit TPCs. Importantly, our findings support the temperature–size rule in arthropods (Atkinson 1995), including mosquitoes (Huxley et al. 2022; Agyekum et al. 2022), where adult body size decreases with increasing developmental temperature.

Although the temperature dependence of juvenile traits is well‐established, much less is known about how these responses are shaped by humidity (reviewed in Brown et al. 2023). A small number of laboratory studies across insect taxa have explicitly examined the combined effects of temperature and relative humidity on performance, survival or development, often reporting strong but context‐dependent interactions (e.g., Gol'berg 1982; Yadav and Chaudhary 1986; Rocha et al. 2001; Simelane 2007; Chiarelli et al. 2011; Kumar et al. 2013; Sauer et al. 2022). However, this literature has focused almost exclusively on terrestrial life stages or adult traits, and typically treats humidity as a modifying stressor rather than as a factor that reshapes thermal performance curves or population‐level outcomes. Consequently, how temperature–humidity interactions structure juvenile trait performance and downstream population fitness in insects with aquatic life stages has remained largely unexplored.

In this study, we found that reduced humidity shifted the thermal performance of juvenile survival and development toward warmer temperatures. The negative relationship between temperature and adult body size also weakened under reduced humidity. Together, these results indicate that higher RH can steepen thermal penalties at warm temperatures for juvenile survival and adult size, whereas lower RH shifts juvenile thermal responses toward warmer temperatures. Such effects may shape seasonal and spatial variation in aquatic habitat productivity and mosquito population fitness, with potential downstream consequences for transmission dynamics (Ameneshewa and Service 1996; Moller‐Jacobs et al. 2014; Shapiro et al. 2016; Lemos‐Silva et al. 2025). Humidity effects on juvenile traits, especially survival, strongly influenced $r_{m}$, with effects most pronounced when evaporation was high. Under partial evaporation conditions (≥60% RH), declining water volume may also increase the concentration of available food, dissolved residues or metabolic by‐products, which could contribute to the observed effects of relative humidity on juvenile survival, development time, body size upon emergence and predicted population growth rates alongside direct temperature–humidity interactions. Field studies in *Aedes albopictus* support these findings, showing that higher daily mean humidity reduces adult emergence and population growth during warm seasons (Murdock et al. 2017).

The non‐monotonic responses we observed—where intermediate relative humidity maximized juvenile performance—suggest that both low and high humidity impose distinct physiological constraints, and that intermediate conditions may represent a balance among competing processes affecting aquatic habitats. Below, we propose three non‐mutually exclusive mechanisms by which temperature and humidity interact to influence juvenile life history traits and $r_{m}$. First, both alter surface tension: high temperatures reduce it, while higher humidity further lowers it by reducing evaporation (Pérez‐Díaz et al. 2012). In cool, dry environments, elevated surface tension may increase the metabolic costs of diving and accessing atmospheric oxygen, whereas in hot, humid environments, very low surface tension may hinder successful emergence (Bush et al. 2007). Mortality under surface‐tension extremes has been reported across mosquito species (Singh and Micks 1957). Second, temperature and humidity affect oxygen concentration in both air and water (Koue 2024). Warm, humid air holds less oxygen, potentially constraining juveniles that rely on atmospheric oxygen (Mamai et al. 2016; Ha et al. 2017). Similarly, warm water holds less dissolved oxygen, limiting diving, feeding and predator avoidance (Clements 1992; Futami et al. 2008). However, work on other insects suggests that oxygen must be drastically limited to strongly constrain survival (Harrison and Haddad 2011). Third, faster evaporation under low humidity may cool water and increase dissolved oxygen at the surface (Kurose et al. 2009; Fukatani et al. 2016), potentially allowing larvae to tolerate higher ambient temperatures by surviving better, developing faster and emerging larger relative to conspecifics developing in more humid environments.

Incorporating humidity into thermal performance models for *An. stephensi* substantially altered predictions of climate suitability across India and Africa (Figure 5; see Figure S9 for maps showing seasonal mean temperatures and relative humidity for 1970–2000 across Africa and India). Consistent with these spatial shifts, the temperature‐only model predicted larger areas of year‐round suitability ($r_{m}>0$ in all months) than the temperature × humidity model: by ∼0.4 million km^2^ in South Asia (3.1 vs. 2.7 million km^2^) and ∼0.8 million km^2^ in Africa (25.0 vs. 24.2 million km^2^). Seasonal patterns also shifted, with models including humidity predicting greater suitability during rainy seasons, while temperature‐only models favoured drier seasons. Across both regions, mean temperatures remain below the thermal optima for $r_{m}$ regardless of humidity, but suitability may persist above ∼35°C if relative humidity simultaneously declines. These maps should be viewed as heuristic, but they highlight how omitting humidity could misidentify seasonal windows of environmental suitability or introduce spatial biases between hot–dry and warm–humid regions.

This study has several limitations. First, due to export laws in countries where *An. stephensi* is native, our experiments were conducted under laboratory conditions using a single, long‐established colony. Laboratory colonies may experience founder effects, inbreeding, drift or laboratory adaptation (Ross et al. 2019; Gloria‐Soria et al. 2019) and environmental–trait relationships may therefore differ quantitatively from those of locally adapted field populations (Dennington et al. 2024; Couper et al. 2025). Our objective, however, was not to estimate population‐specific demographic parameters across the species range, but to isolate the qualitative structure of temperature–humidity interactions under controlled conditions. Second, our experiments were conducted under constant temperature and humidity. Such designs simplify natural environments, but they are the standard basis for estimating thermal performance relationships and for isolating the contribution of individual environmental drivers under controlled settings (Sinclair et al. 2016). Extending these results to fluctuating thermal and humidity regimes is an important next step, especially because realized juvenile and adult environments in nature depend on microclimate, habitat structure and behaviour. Third, our climate suitability metric, $r_{m}$, did not explicitly include humidity effects on adult mortality and fecundity, which may be influenced both directly (Brown et al. 2023) and indirectly through carry‐over effects from juvenile stages (Moller‐Jacobs et al. 2014; Shapiro et al. 2016). Because adult trait responses and habitat availability are not included, the spatial analyses should be interpreted as relative contrasts between model structures rather than estimates of realized mosquito abundance or range. Fourth, habitat availability—shaped by precipitation, water access, storage practices and evaporation—also mediates environmental suitability (Stewart Ibarra et al. 2013; Hayden et al. 2010; Whittaker et al. 2023). While *An. stephensi* often develops in larger containers that are less prone to drying (Thomas et al. 2016), other vectors use smaller, more ephemeral habitats that are more sensitive to evaporation (Day 2016). Although we standardized juvenile resource availability, temperature and humidity may indirectly influence microbial growth and food quality, thereby altering competition and $r_{m}$ (Guégan et al. 2018; Coon et al. 2016; Takken et al. 2013; White et al. 2011).

There are several lines of evidence that support cautious generalization of these results. While local adaptation can shift thermal performance parameters quantitatively, it does not generally alter the qualitative form of temperature–trait relationships in mosquitoes (Mordecai et al. 2019; Dennington et al. 2024) or in other ectotherms (Chown and Nicolson 2004). More broadly, experimental work in other insect systems demonstrates that relative humidity can modify temperature‐dependent performance in stage‐ and species‐specific ways, reinforcing the expectation that temperature–humidity interactions are biologically real but contingent rather than universal (Gol'berg 1982; Yadav and Chaudhary 1986; Rocha et al. 2001; Simelane 2007; Chiarelli et al. 2011; Kumar et al. 2013; Sauer et al. 2022). Finally, our results align with field studies of other mosquito vectors highlighting the importance of microclimate and humidity for population dynamics (Murdock et al. 2017; Evans et al. 2019), as well as epidemiological work on the *An. stephensi*–malaria system (Santos‐Vega et al. 2022). This supports cautious generalization of the existence and qualitative importance of temperature–humidity interactions while avoiding overstatement about species‐wide demographic parameterisation. While our study represents a foundational first step, further work with natural populations is needed.

In conclusion, our results align with recent conceptual work showing that temperature–humidity interactions determine whether mortality is driven primarily by heat stress or by water‐balance constraints, and that the threshold between these regimes can shift predictably with environmental conditions (Sinclair et al. 2024). Importantly, our study extends this framework to aquatic juvenile systems, demonstrating that relative humidity can indirectly influence thermal vulnerability through evaporation dynamics and air–water interface processes that restructure aquatic rearing environments. Relative humidity also altered the temperature–size relationship in adults. Separately, humidity‐driven changes in juvenile survival and development resulted in systematic shifts in projected $r_{m}$. More broadly, our findings show that incorporating humidity alongside temperature can change trait‐based inference about environmental suitability in a medically important insect, providing a mechanistic basis for extending thermal performance frameworks to include additional environmental drivers in mosquitoes and other temperature‐sensitive species.

## Author Contributions

C.C.M., L.R.J. and P.J.H. conceived the study and designed the experiments. J.J.B., B.S.L., B.J., O.Y.C. and A.A. performed the experiments and collected the data. M.C.W. and E.R.B. created the maps. P.J.H., L.R.J. and B.D.H. performed statistical analyses and modelling. P.J.H. wrote the first draft of the manuscript, and all authors contributed substantially to revisions.

## Funding

This work was supported by R01AI153444‐03S1, R01AI163444.

## Supporting information


**Figure S1:** Observed juvenile survival data (hatching‐to‐adult) across temperature–humidity levels. There were *n* = 3 replicates per treatment each containing *n* = 100 L1 larvae at the start of the experiment. The figure shows the number of survivors for each treatment from a pooled total of *n* = 300 at the start of the experiment. No individuals survived to adulthood at 14°C and 42°C irrespective of humidity level.
**Figure S2:** Juvenile development time TPCs (hatching‐to‐adult) used for the temperature‐ and humidity‐dependent *r*_m_ calculations. Development time (α in Equation 1, Main text) TPCs were fitted using Equation SE1. Points are individual mosquitoes. Relative humidity (%) levels are shown in the title boxes.
**Figure S3:** Robustness of *r*_m_ thermal limits and optima to ±25% and ±50% adult‐constant scaling. Comparison of baseline and perturbed estimates of *T*_min_, *T*_max_, *T*_opt_ and *r*_opt_ across relative humidity (RH) treatments under controlled evaporation. Points show medians; bars show 95% HPD intervals. Perturbations simultaneously scale adult mortality (z) and fecundity (*b*_max_) constants by ±25% and ±50% using the same posterior fits for juvenile traits (α and *p*_EA_). Shifts in all parameter estimates are modest and do not alter qualitative humidity‐driven patterns, indicating *r*_m_'s temperature dependence is primarily driven by juvenile survival with limited sensitivity to adult‐constant scaling.
**Figure S4:** Uncontrolled evaporation: Relative humidity shapes the temperature dependence of juvenile fitness traits in An. stephensi (a–f). a. Humidity–dependent survival probability TPCs (*p*_EA_ in Equation 2, Main text). b and c. b. Numerical survival probability parameter estimates of *T*_min_ and *T*_max_ (Table S8). c. Predicted peak survival probabilities at *T*_pk_ at each humidity level (Table S9). Legend in b also applies to c. d. Humidity‐dependent development rate TPCs (1/α in Equation 2). e. Development rate parameter estimates for *T*_min_ versus *T*_max_ across humidity levels (Table S11). f. Predicted peak development rate at *T*_pk_ at each humidity level (Table S10). Prediction bounds in a and d are HPD intervals estimated from the posteriors for each TPC. In b & c and e & f, error bars represent 95% credible intervals summarizing posterior uncertainty.
**Figure S5:** Uncontrolled evaporation: Effects of relative air humidity on juvenile fitness traits shape the temperature dependence of maximal population growth rate, *r*_m_. (a–c) a. *r*_m_ TPCs across relative humidity levels. (b and c) b. *r*_opts_ versus *T*_opts_ (Table S12), c. *T*_min_ versus *T*_max_ across humidity levels (Table S13). Prediction bounds in a are HPD intervals calculated using the posteriors for each humidity‐dependent TPC. Error bars in b and c represent 95% credible intervals summarizing posterior uncertainty.
**Figure S6:** Uncontrolled evaporation: Relative air humidity and temperature interact to modulate the temperature–size relationship in Anopheles stephensi. Regression lines show that body size decreased with temperature (Slope: −0.10998; CrI: −0.1397837, −0.0805072) and humidity (Slope: −0.0301336; CrI: −0.0412171, −0.0191704), and body size decreased more steeply with temperature at lower humidity (Interaction: 0.0007560; CrI: 0.0004171, 0.0010998). Boxplot horizontal lines represent medians; lower and upper hinges are the 25th and 75th percentiles. Upper whiskers extend from the hinge to the largest value no further than 1.5 × inter‐quartile range (IQR) from the hinge. The lower whisker extends from the hinge to the smallest value at most 1.5 × IQR of the hinge. Points represent individual mosquitoes.
**Figure S7:** Uncontrolled evaporation: Observed juvenile survival data (hatching–to–adult) across temperature–humidity levels. There were *n* = 3 replicates per treatment each containing *n* = 100 L1 larvae at the start of the experiment. The figure shows the number of survivors for each treatment from a pooled total of *n* = 300 at the start of the experiment. No individuals survived to adulthood at 14°C and 42°C irrespective of the humidity level.
**Figure S8:** Uncontrolled evaporation: Juvenile development time TPCs (hatching–to–adult) used for the temperature‐ and humidity‐dependent *r*_m_ TPCs. Development time (α in Equation 2) TPCs were fitted using Equation SE1. Points are individual mosquitoes.
**Figure S9:** Seasonal mean temperatures and relative humidity across Africa and South Asia under historical climate conditions (1970–2000). Panels (a) and (c) show seasonal mean temperatures across Africa and India for 1970–2000, respectively. Panels (b) and (d) show mean annual relative humidity by season across Africa and India for the same period, respectively.
**Table S1:** Block‐wise assignment of temperature, relative humidity, incubator ID, colony generation, evaporation setting (C = controlled, E = uncontrolled), number of trays per treatment and starting number of individual mosquitoes per tray (N0).
**Table S2:** Estimates for the juvenile survival TPC parameters; *B*_pk_ and *T*_pk_. Numerical posterior estimates (median ±95% credible intervals) of the parameters from *n* = 10,000 iterations for a burn–in of *n* = 5000.
**Table S3:** Estimates for the juvenile survival TPC parameters; *T*_min_ and *T*_max_. Numerical posterior estimates (median ±95% credible intervals) of the parameters from *n* = 10,000 iterations for a burn–in of *n* = 5000.
**Table S4:** Estimates for the juvenile development rate TPC parameters; *B*_pk_ and *T*_pk_. Numerical posterior estimates (median ±95% credible intervals) of the parameters from *n* = 10,000 iterations for a burn–in of *n* = 5000.
**Table S5:** Estimates for the juvenile development rate TPC parameters; *T*_min_ and *T*_max_. Posterior estimates (mean ±95% credible intervals) of the standard Briere model (SE2) parameters from *n* = 10,000 iterations for a burn–in of *n* = 5000.
**Table S6:** Estimates for the *r*_m_ TPC parameters; *T*_opts_ and *r*_opt_. Numerical posterior estimates (median ±95% credible intervals) of the *r*_m_ model (Equation 1, Main text) parameters from *n* = 10,000 iterations for a burn–in of *n* = 5000.
**Table S7:** Estimates for the *r*_m_ TPC parameters; *T*_min_ and *T*_max_. Numerical posterior estimates (median ±95% credible intervals) of the parameters from *n* = 10,000 iterations for a burn–in of *n* = 5000.
**Table S8:** Estimates for the juvenile survival TPC parameters; *B*_pk_ and *T*_pk_. Numerical posterior estimates (median ±95% credible intervals) of the parameters from *n* = 10,000 iterations for a burn–in of *n* = 5000.
**Table S9:** Estimates for the juvenile survival TPC parameters; *T*_min_ and *T*_max_. Numerical posterior estimates (median ±95% credible intervals) of the parameters from *n* = 10,000 iterations for a burn–in of *n* = 5000.
**Table S10:** Estimates for the juvenile development rate TPC parameters; *B*_pk_ and *T*_pk_. Numerical posterior estimates (median ±95% credible intervals) of the parameters from *n* = 10,000 iterations for a burn–in of *n* = 5000.
**Table S11:** Estimates for the juvenile development rate TPC parameters; *T*_min_ and *T*_max_. Posterior estimates (mean ±95% credible intervals) of the standard Briere model (SE2) parameters from *n* = 10,000 iterations for a burn–in of *n* = 5000.
**Table S12:** Estimates for the *r*_m_ TPC parameters; *T*_opts_ and *r*_opt_. Numerical posterior estimates (median ±95% credible intervals) of the parameters from *n* = 10,000 iterations for a burn–in of *n* = 5000.
**Table S13:** Estimates for the *r*_m_ TPC parameters; *T*_min_ and *T*_max_. Numerical posterior estimates (median ±95% credible intervals) of the parameters from *n* = 10,000 iterations for a burn–in of *n* = 5000.

## Data Availability

All data and code supporting this study are archived in Dryad and are available for review at: https://doi.org/10.5061/dryad.6q573n6db. The data is also deposited in VecTraits, a fully open, machine‐readable database for arthropod vector traits.
