# Supplementary material for: Beyond Temperature: Relative Humidity Systematically Shifts Juvenile Thermal Performance and Projected Population Growth in a Malaria Vector
Source: Ecol Lett. 2026 Jun 11;29(6):e70416. doi: 10.1111/ele.70416 (PMC13261101; doi:10.1111/ele.70416)
Supplement: Supplementary file 1 — Figure S1: Observed juvenile survival data (hatching‐to‐adult) across temperature–humidity levels. There were n = 3 replicates per treatment each containing n = 100 L1 larvae at the start of the experiment. The figure shows the number of survivors for each treatment from a pooled total of n = 300 at the start of the experiment. No individuals survived to adulthood at 14°C and 42°C irrespective of humidity level. Figure S2: Juvenile development time TPCs (hatching‐to‐adult) used for the temperature‐ and humidity‐dependent r m calculations. Development time (α in Equation 1, Main text) TPCs were fitted using Equation SE1. Points are individual mosquitoes. Relative humidity (%) levels are shown in the title boxes. Figure S3: Robustness of r m thermal limits and optima to ±25% and ±50% adult‐constant scaling. Comparison of baseline and perturbed estimates of T min, T max, T opt and r opt across relative humidity (RH) treatments under controlled evaporation. Points show medians; bars show 95% HPD intervals. Perturbations simultaneously scale adult mortality (z) and fecundity (b max) constants by ±25% and ±50% using the same posterior fits for juvenile traits (α and p EA). Shifts in all parameter estimates are modest and do not alter qualitative humidity‐driven patterns, indicating r m's temperature dependence is primarily driven by juvenile survival with limited sensitivity to adult‐constant scaling. Figure S4: Uncontrolled evaporation: Relative humidity shapes the temperature dependence of juvenile fitness traits in An. stephensi (a–f). a. Humidity–dependent survival probability TPCs (p EA in Equation 2, Main text). b and c. b. Numerical survival probability parameter estimates of T min and T max (Table S8). c. Predicted peak survival probabilities at T pk at each humidity level (Table S9). Legend in b also applies to c. d. Humidity‐dependent development rate TPCs (1/α in Equation 2). e. Development rate parameter estimates for T min versus T max across humidity levels [file ELE-29-0-s001.pdf]

Supporting Information for

Beyond temperature: Relative humidity systematically shifts juvenile thermal performance and projected population growth in a malaria vector

Experimental Design

Table S1: Block-wise assignment of temperature, relative humidity, incubator ID, colony generation, evaporation setting (C=controlled, E=uncontrolled), number of trays per treatment, and starting number of individual mosquitoes per tray (N_0).

**Block**	**Temperature (°C)**	**RH (%)**	**Incubator (ID)**	**Colony generation**	**Evaporation**	**Trays (n)**	N_0
1	16	60	I1	F11	C/E	3	100
1	20	60	I2	F11	C/E	3	100
1	24	60	I3	F11	C/E	3	100
1	28	45	I4	F11	C/E	3	100
1	32	45	I5	F11	C/E	3	100
1	35	75	I6	F11	C/E	3	100
1	38	30	I7	F11	C/E	3	100
2	16	90	I8	F13	C/E	3	100
2	20	30	I2	F13	C/E	3	100
2	24	45	I3	F13	C/E	3	100
2	28	60	I4	F13	C/E	3	100
2	32	75	I5	F13	C/E	3	100
2	35	60	I6	F13	C/E	3	100
2	38	90	I7	F13	C/E	3	100
3	16	30	I1	F15	C/E	3	100
3	20	45	I2	F15	C/E	3	100
3	24	75	I3	F15	C/E	3	100
3	28	75	I4	F15	C/E	3	100
3	32	90	I5	F15	C/E	3	100
3	35	30	I6	F15	C/E	3	100
3	38	60	I8	F15	C/E	3	100
4	16	45	I8	F17	C/E	3	100
4	20	90	I2	F17	C/E	3	100
4	24	30	I3	F17	C/E	3	100
4	28	30	I4	F17	C/E	3	100
4	32	60	I9	F17	C/E	3	100
4	35	90	I5	F17	C/E	3	100
4	38	75	I6	F17	C/E	3	100
5	16	75	I1	F20	C/E	3	100
5	20	75	I4	F20	C/E	3	100
5	24	90	I3	F20	C/E	3	100
5	28	90	I9	F20	C/E	3	100
5	32	30	I5	F20	C/E	3	100
5	35	45	I6	F20	C/E	3	100
5	38	45	I7	F20	C/E	3	100
6	40	60	I6	F21	C/E	3	100
7	40	30	I5	F21	C/E	3	100
7	40	45	I1	F21	C/E	3	100
7	40	75	I6	F21	C/E	3	100
7	40	90	I7	F21	C/E	3	100

Supplementary Methods

Experimental set-up (detailed)

We used an *An. stephensi* urban–type strain acquired from a longstanding (∼40 years) colony at Walter Reed Army Institute of Research via the University of Georgia due to export restrictions for biological materials in the native range (India). Mosquitoes were maintained using established methods ((Miazgowicz et al., 2020; Pathak et al., 2019)).Using environmental chambers (Percival Scientific, 36VL H15 ultrasonic humidification and dehumidification), we exposed immature mosquitoes to eight constant temperatures (16°C, 20°C, 24°C, 28°C, 32°C, 35°C, 38°C, 40°C), five relative humidity levels (30%, 45%, 60%, 75%, and 90% ± 5%), and 12:12 light:dark cycles (Table S1). Additional temperature treatments at 14°C and 42°C were included during the experiment to better characterize the lower and upper thermal limits constraining juvenile mosquito performance. These temperature and relative humidity gradients were chosen to span conditions experienced across the native and invasive range of *An. stephensi* and to characterize physiological limits and non-linear responses.

We also included an uncontrolled evaporation treatment to assess how juvenile trait responses changed when water loss intensified and rearing environments diminished in volume. Larvae were held within incubators in three replicate plastic trays per treatment (Sterilite, 5.7 L; 35 cm × 21 cm × 12 cm), covered with fine mesh. Each tray was seeded with 100 first–instar (L1) larvae, four pellets of Cichlid Gold fish food, and 1 L of purified (reverse osmosis) water pre–equilibrated to the treatment temperature. For controlled–evaporation trays, evaporative water loss was recorded daily and replaced to maintain constant volume; trays were rotated within incubators to minimize spatial heterogeneity in evaporation. Upon pupation, pupae were transferred to 250 mL plastic cups containing treatment–temperature water and placed into 17.5 cm × 17.5 cm × 17.5 cm fine–mesh cages (BugDorm Small) within the corresponding incubator. Data collected from these experiments were used to quantify the effects of relative humidity on juvenile survival probability, development time, body size upon emergence, and maximal population growth rate (r_m) as a metric of population fitness.

Modeling the development time TPCs

We modeled the development time TPCs for each humidity level in two ways. First, to get the juvenile development time (α) for our r_m calculations (Eqn. 2, Main text), we implemented an exponential decay function (SE1) in the `bayesTPC` package in R (Sorek et al., 2025) to directly fit to the observed data for this trait (Figs. S2, S8).

$$\alpha(T) = a^{(-rT)} + c \tag{SE1}$$

Here, a is a scaling factor determining the amplitude of the decay, r is the rate of decay (where r is > 0), T is the temperature in degrees Celsius, and c is a constant that represents the baseline that $\alpha(T)$ approaches as $T \to \infty$.

However, this model (SE1) is not unimodal and does not have defined values for T_{min}, T_{pk}, B_{pk}, and T_{max}. Thus in order to be able to make a more direct comparison to previous studies, we fitted the standard Briere model (SE2; as implemented in the `bayesTPC` package in R; Briere et al., 1999; Sorek et al., 2025) to *inverted development times* (i.e., development rate; $1/\alpha$ in Eqn. 2).

$$1/\alpha(T) = q \cdot T \cdot (T - T_{\mathrm{min}}) \cdot \sqrt{(T_{\mathrm{max}} > T) \cdot (T_{\mathrm{max}} - T)} \cdot (T_{\mathrm{max}} > T) \cdot (T > T_{\mathrm{min}}) \tag{SE2}$$

Here, T is temperature in degrees Celsius, T_{min} is the low temperature (ºC) at which rates become negative, T_{max} is the high temperature (ºC) at which rates become negative, and q is a scale parameter that sets the maximum rate of the curve. The temperatures at which trait performance peaks and its value at its peak (T_{pk} and B_{pk}, respectively; Table 2, Main text) were estimated numerically from the posterior distributions for each humidity level’s TPC and summarized by the posterior medians and Highest Posterior Density (HPD) intervals.

Modelling the survival probability TPCs

The probability of juvenile survival was estimated from emergence data (number emerged from the initial cohort) by fitting humidity-specific thermal performance curves using a binomial generalized linear model with a logit link function (Eqn. SE3), as implemented in the `bayesTPC` package (Sorek et al., 2025). Specifically, we parameterized the probability of juvenile-to-adult survival (p_{EA} in Eqn. 2, Main text) by fitting a quadratic function of temperature to the binomial survival data at each humidity level,

$$p_{\mathrm{EA}}(T) = b_0 + b_1 \cdot T + b_2 \cdot T^2, \tag{SE3}$$

where T is temperature in degrees Celsius, b_0 represents survival probability at $T = 0$, b_1 describes the linear temperature dependence of survival, and b_2 captures curvature in the temperature–survival relationship. As for development rate, we estimated key quantities of interest (T_{pk} and B_{pk}) numerically from the posterior distributions for each humidity-specific TPC. Because the fitted survival curves asymptotically approach, but do not equal, zero, we defined the lower and upper thermal limits (T_{min} and T_{max}) at each humidity level as the temperatures at which predicted survival fell to $\leq 1\%$.

Modeling temperature–size relationships

Wing length was measured as a proxy for body size and fecundity. Both relationships are expected to be linear—larger females have longer wings, greater body mass, and are more fecund (Briegel, 1990a,b). To analyze how relative humidity can affect the temperature–size rule (i.e., body size generally decreases with temperature in ectotherms; Atkinson, 1995), we fit a Bayesian hierarchical regression model to adult female wing lengths using the `brms` package in R (Bürkner, 2017; R Core Team, 2023). In this model (WingLength $\sim$ Temp $\times$ RH + (1|Tray)), wing length is the outcome variable and temperature and relative humidity (RH) are the predictor variables; the random intercept for tray accounts for shared rearing conditions within trays. The interaction term (Temp $\times$ RH) tests whether humidity modifies the temperature–size relationship. As a robustness check aligned with the blocked design, we refit this model with random intercepts for Block and trays nested within Block, (1|Block) + (1|Block:Tray); fixed-effect estimates were unchanged within 95% credible intervals (see Results; SM). The model assumes a Gaussian distribution and was fit for 5000 iterations using Hamiltonian Monte Carlo with a maximum tree depth of 20.

To quantify the relationship between development time and adult size, we fit additional models at both the tray and individual level. At the tray level, we regressed mean wing length on tray mean development time, temperature, RH, and their interaction, providing a cohort-level description of size–development relationships under shared rearing conditions. At the individual level, we fit a hierarchical Bayesian regression for wing length with predictors mean development time, RH, temperature, their interaction (mean development time $\times$ RH), and random intercepts for both Block and trays nested within Block (WingLength $\sim$ mean_dt $\times$ RH + Temp + (1|Block) + (1|Block:Tray)). Together, these models test whether development time predicts adult size and whether this relationship is modulated by humidity, while appropriately accounting for hierarchical sampling and shared environmental history.

Trait sensitivity analysis

To determine the extent to which variation in humidity can affect the relative contributions of the juvenile fitness traits to the temperature dependence of r_m, using the chain rule, we can write (Cator et al., 2020):

$$\frac{\mathrm{d}r_\mathrm{m}}{\mathrm{d}T} = \frac{\partial r_\mathrm{m}}{\partial b_\mathrm{max}}\frac{\mathrm{d}b_\mathrm{max}}{\mathrm{d}T} + \frac{\partial r_\mathrm{m}}{\partial \alpha}\frac{\mathrm{d}\alpha}{\mathrm{d}T} + \frac{\partial r_\mathrm{m}}{\partial z}\frac{\mathrm{d}z}{\mathrm{d}T} + \frac{\partial r_\mathrm{m}}{\partial p_\mathrm{EA}}\frac{\mathrm{d}p_\mathrm{EA}}{\mathrm{d}T} + \frac{\partial r_\mathrm{m}}{\partial \kappa}\frac{\mathrm{d}\kappa}{\mathrm{d}T}. \tag{SE4}$$

Each summed term on the right-hand side of this equation quantifies the relative contribution of each trait TPC parameter in Eqn. 2 (Main text) to the temperature dependence of r_m. To calculate the derivatives associated with each term in Eqn. SE4, we used the MAP (Maximum A Posteriori) estimator for each

trait–humidity combination (Figs. 4 (Main text) & S4). The sample-based MAP estimator is calculated as part of the MCMC fitting process in the `bayesTPC` package (Sorek et al., 2025) in R. We used this method to estimate the Eqn. 2 parameters because it maximizes the posterior probability of the unknown parameter given the observed data and prior beliefs and, unlike the Maximum Likelihood Estimator (MLE), it incorporates prior information.

Robustness checks for constant adult trait assumption

As noted in the Main text, to isolate humidity's effects on juvenile traits, we held adult mortality (z) and fecundity (b_{max}) constant in the r_{m} formulation; in the trait-sensitivity decomposition, adult terms contribute to $\mathrm{d}r_{\mathrm{m}}/\mathrm{d}T$ only through their temperature derivatives, which are zero under this assumption. As a robustness check using the same posterior fits for juvenile traits (α and p_{EA}), we recomputed r_{m} while *simultaneously* scaling the adult constants (b_{max} and z) by $\pm 25\%$ and $\pm 50\%$, and we report the resulting changes in T_{min}, T_{max}, T_{opt}, and r_{opt} relative to baseline. The overlays and summaries are shown below in Fig. S3, alongside the baseline in Fig. 3b,c (Main text).

Methods for Mapping Study Region and Data Sources

We mapped the maximal mosquito population growth rate (r_{m}) across South Asia and Africa under historical climate conditions. The South Asian study region encompassed India and its neighboring countries (Bangladesh, Bhutan, Nepal, Sri Lanka, Pakistan, Maldives, and Afghanistan), capturing diverse climates from tropical to temperate zones. The African study region covered the entire continent, incorporating equatorial, savannah, arid, and subtropical climates to enable broad regional comparisons of potential mosquito growth. Climate variables, including daily maximum temperature, minimum temperature, and specific humidity, were obtained from the NASA NEX–GDDP–CMIP6 dataset. These statistically downscaled, bias–corrected projections have a resolution of $0.25^{\circ} \times 0.25^{\circ}$ ($\sim$27.8 km). We incorporated outputs from 23 general circulation models (GCMs) in this dataset, analyzing the ensemble of historical (1970–2000) model runs to generate climate inputs for the models.

Climate Data Processing

We used the NASA climate data to estimate r_{m}, which integrates the effects of temperature and humidity on mosquito population dynamics. Daily climate variables including maximum temperature, minimum temperature, and specific humidity from 1970 to 2000 were masked to terrestrial grid cells within the study–region boundaries. These daily data were first aggregated into monthly averages. Monthly mean temperatures

were calculated as the average of daily maximum and minimum temperatures. Relative humidity was computed from monthly averaged specific humidity data using the Magnus–Tetens equation, accounting for temperature-dependent saturation vapor pressure. Finally, seasonal means were calculated by averaging monthly data across defined three–month periods (e.g., January–March) for the entire 1970–2000 period.

Trait-Based Population Growth Model

Monthly mosquito population growth was predicted using the trait-based projection model of maximal population growth rate (r_m). Juvenile traits (survival probability and developmental rate) were modeled as nonlinear functions of temperature based on laboratory experiments. Temperature–trait curves were derived from the experimental RH values of 30%, 45%, 60%, 75%, and 90%. To quantify the influence of humidity, we conducted two model runs. The temperature-only model held humidity constant at a fixed reference value of 75% RH, approximating a mid-range humidity level within the experimental design. The temperature $\times$ RH model used the actual RH values from the climate dataset. We applied piecewise linear interpolation to handle continuous RH variations in the climate data, deriving each modeled trait from the two nearest experimental RH levels. This method ensured smooth trait responses to varying humidity without abrupt category transitions. These analyses produced monthly raster datasets of r_m values for the two model runs, and the monthly outputs were summarized to generate mean annual r_m.

Spatial Analysis and Mapping

Maps of mean annual projected r_m were generated to highlight spatial variability in mosquito population growth potential. Difference maps (Δr_m) were created to highlight areas where adding humidity altered the growth rates, illustrating regions where the inclusion of humidity alters projected suitability for population growth. We also computed the total land area for which climate was suitable for mosquito growth ($r_\mathrm{m} > 0$) during all months of the year (Fig. S9).

Supplementary Results

Temperature, humidity, and body size

We observed significant effects of temperature, relative humidity (RH), and their interaction on adult size upon emergence (wing length, Figure 2, Main text). Generally, increasing temperature decreased body size (Slope: -0.0216; 95% CrI: -0.0276, -0.0158), while increasing humidity tended to increase it (Slope: 0.0063; 95% CrI: $-0.0034, 0.0092$). However, as RH increased, the negative effect of temperature on

body size became steeper, with steeper declines in body size upon emergence with increasing temperature (interaction: −0.0002; 95% CrI: −0.0003, −0.0001). As temperatures increased from 16 to 40 °C, size was predicted to decrease by ∼1 mm at 90% RH, whereas at 30% RH it was predicted to decrease by ∼0.07 mm.

In habitats subject to evaporation, mosquitoes in general had larger wing sizes upon emergence (Evaporation uncontrolled: 2.93 mm ± 0.314 (mean ± SD, $n = 678$); Evaporation controlled: 2.81 mm ± 0.318 ($n = 932$)). Further, while the effects of temperature and relative humidity were similar to environments experiencing minimal evaporation, the interaction effect was stronger across a smaller range of relative humidity (Figure S6). Size decreased both at warmer temperatures (Slope: −0.110; 95% CrI: −0.140, −0.081) and at lower humidity levels (Slope: −0.030; 95% CrI: −0.041, −0.019), but in contrast with the controlled treatments (where higher RH steepened the temperature–size decline), the decrease with temperature was greater at lower humidity levels (75 and 60% RH) than at the highest humidity level (90%; interaction: 0.00076; 95% CrI: 0.00042, 0.00110). As temperatures increased from 16 to 40 °C, size was predicted to decrease by ∼1.43 mm at 60% RH, whereas at 90% RH it decreased by ∼0.92 mm (Figure S6). These results are robust to the hierarchical sampling structure: refitting wing length with random intercepts for Block and trays nested within Block yielded fixed-effect estimates that overlapped those from the tray-only model within 95% credible intervals (Temp, RH, Temp×RH), and therefore do not change the substantive conclusion (see Figure 2, Main text).

To test whether development time predicts adult size and how this relationship is moderated by RH, we fit both tray-level and hierarchical models. At the tray level, mean development time and mean wing length were strongly positively correlated within RH strata (e.g., $r = 0.819$ at 30% RH; $r = 0.808$ at 90% RH), reflecting their shared temperature dependence. An adjusted tray-level regression (*wl_mean* ∼ *mean_dt* + *Temp* + *RH* + *Temp:RH*) found a modest positive slope for development time (+0.0055 mm per day; 95% CrI: −0.0005, +0.0114), a strong negative temperature effect (−0.025 mm/°C; 95% CrI: −0.0335, −0.0165), and steeper temperature–size declines at some higher RH (significant Temp×RH at 60% and 90%). An individual-level hierarchical model controlling for temperature and sampling structure (*WingLength* ∼ *mean_dt* × *RH* + *Temp* + *(1|Block)* + *(1|Block:Tray)*) found the mean_dt×RH interaction term was positive (0.000121 mm·day^{-1}·%RH^{-1}; 95% CrI 0.000011, 0.000230), indicating that the temperature-adjusted association between development time and size becomes more positive at higher RH. Posterior simple slopes (mm per day) were: 30% RH −0.0045 (Pr>0 = 0.08), 45% RH −0.0027 (0.16), 60% RH −0.0009 (0.35), 75% RH +0.0009 (0.65), and 90% RH +0.0027 (0.86). In summary, after adjusting for temperature, the development time–size link is near zero at low RH and increasingly positive at higher RH, consistent with the panelled trends and within-RH point clouds in Figure 2 (Main text).

174 ## Supplementary figures and tables

Table S2: Estimates for the juvenile survival TPC parameters; B_{pk} and T_{pk}. Numerical posterior estimates (median ± 95% credible intervals) of the parameters from n = 10,000 iterations for a burn–in of n = 5000.

RH (%)	Bpk median	Bpk lower	Bpk upper	Tpk median	Tpk lower	Tpk upper
90	0.937	0.924	0.948	26.176	26.026	26.326
75	0.918	0.903	0.932	28.629	28.428	28.829
60	0.901	0.885	0.916	28.829	28.629	29.029
45	0.835	0.815	0.855	29.279	29.029	29.479
30	0.893	0.876	0.909	30.731	30.531	30.931

Table S3: Estimates for the juvenile survival TPC parameters; T_{min} and T_{max}. Numerical posterior estimates (median ± 95% credible intervals) of the parameters from n = 10,000 iterations for a burn–in of n = 5000.

RH (%)	Tmin	Tmin lower	Tmin upper	Tmax	Tmax lower	Tmax upper
90	12.312	11.962	12.663	39.990	39.640	40.240
75	14.164	13.714	14.565	43.093	42.693	43.493
60	14.665	14.214	15.065	42.993	42.593	43.343
45	13.063	12.513	13.564	45.495	44.945	45.946
30	16.316	15.766	16.717	45.195	44.745	45.596

Table S4: Estimates for the juvenile development rate TPC parameters; B_{pk} and T_{pk}. Numerical posterior estimates (median ± 95% credible intervals) of the parameters from n = 10,000 iterations for a burn–in of n = 5000.

RH (%)	Bpk median	Bpk lower	Bpk upper	Tpk median	Tpk lower	Tpk upper
90	0.141	0.134	0.146	36.236	34.885	36.987
75	0.144	0.140	0.148	36.436	35.586	37.187
60	0.150	0.144	0.155	37.187	36.687	37.487
45	0.142	0.139	0.145	37.287	36.887	37.538
30	0.132	0.125	0.138	37.437	36.887	37.788

Table S5: Estimates for the juvenile development rate TPC parameters; T_{min} and T_{max}. Posterior estimates (mean ± 95% credible intervals) of the standard Briere model (SE2) parameters from n = 10,000 iterations for a burn–in of n = 5000.

RH (%)	Tmin	Tmin lower	Tmin upper	Tmax	Tmax lower	Tmax upper
90	8.653	6.755	10.611	43.901	42.279	45.000
75	11.275	9.832	12.911	43.855	42.765	44.992
60	11.513	9.460	13.687	44.744	44.277	45.000
45	12.392	11.311	13.496	44.699	44.209	45.000
30	13.594	11.827	15.000	44.664	44.060	45.000

Table S6: Estimates for the r_m TPC parameters; T_{opt} and r_{opt}. Numerical posterior estimates (median ± 95% credible intervals) of the r_m model (Eqn. 1, Main text) parameters from n = 10,000 iterations for a burn–in of n = 5000.

RH (%)	ropt median	ropt lower	ropt upper	Topt	Topt lower	Topt upper
90	0.274	0.271	0.278	31.481	31.281	31.682
75	0.286	0.283	0.289	33.834	33.634	34.084
60	0.281	0.279	0.284	33.483	33.233	33.684
45	0.274	0.271	0.277	34.484	34.284	34.735
30	0.270	0.267	0.273	36.086	35.786	36.286

Table S7: Estimates for the r_m TPC parameters; T_{min} and T_{max}. Numerical posterior estimates (median ± 95% credible intervals) of the parameters from n = 10,000 iterations for a burn–in of n = 5000.

RH (%)	Tmin	Tmin lower	Tmin upper	Tmax	Tmax lower	Tmax upper
90	12.462	12.062	12.763	39.890	39.590	40.140
75	14.314	13.814	14.665	42.993	42.593	43.393
60	14.815	14.314	15.215	42.843	42.442	43.193
45	13.263	12.713	13.714	45.345	44.845	45.796
30	16.466	15.916	16.867	45.045	44.645	45.445

Figure S1: Observed juvenile survival data (hatching–to–adult) across temperature–humidity levels. There were n=3 replicates per treatment each containing n=100 L1 larvae at the start of the experiment. The figure shows the number of survivors for each treatment from a pooled total of n=300 at the start of the experiment. No individuals survived to adulthood at 14°C and 42°C irrespective of humidity level.

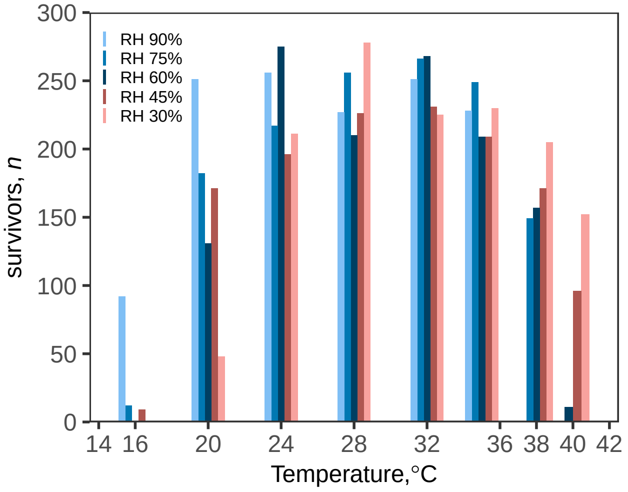

Figure S2: Juvenile development time TPCs (hatching–to–adult) used for the temperature– and humidity–dependent r_{m} calculations. Development time (α in Eqn. 1, Main text) TPCs were fitted using Eqn. SE1. Points are individual mosquitoes. Relative humidity (%) levels are shown in the title boxes.

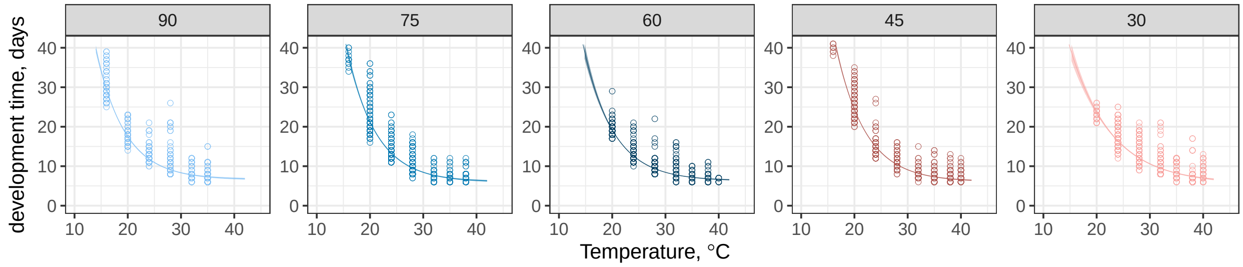

Figure S3: Robustness of r_{m} thermal limits and optima to ±25% and ±50% adult-constant scaling. Comparison of baseline and perturbed estimates of T_{min}, T_{max}, T_{opt}, and r_{opt} across relative humidity (RH) treatments under controlled evaporation. Points show medians; bars show 95% HPD intervals. Perturbations simultaneously scale adult mortality (z) and fecundity (b_{max}) constants by ±25% and ±50% using the same posterior fits for juvenile traits (α and p_{EA}). Shifts in all parameter estimates are modest and do not alter qualitative humidity-driven patterns, indicating r_{m}'s temperature dependence is primarily driven by juvenile survival with limited sensitivity to adult-constant scaling.

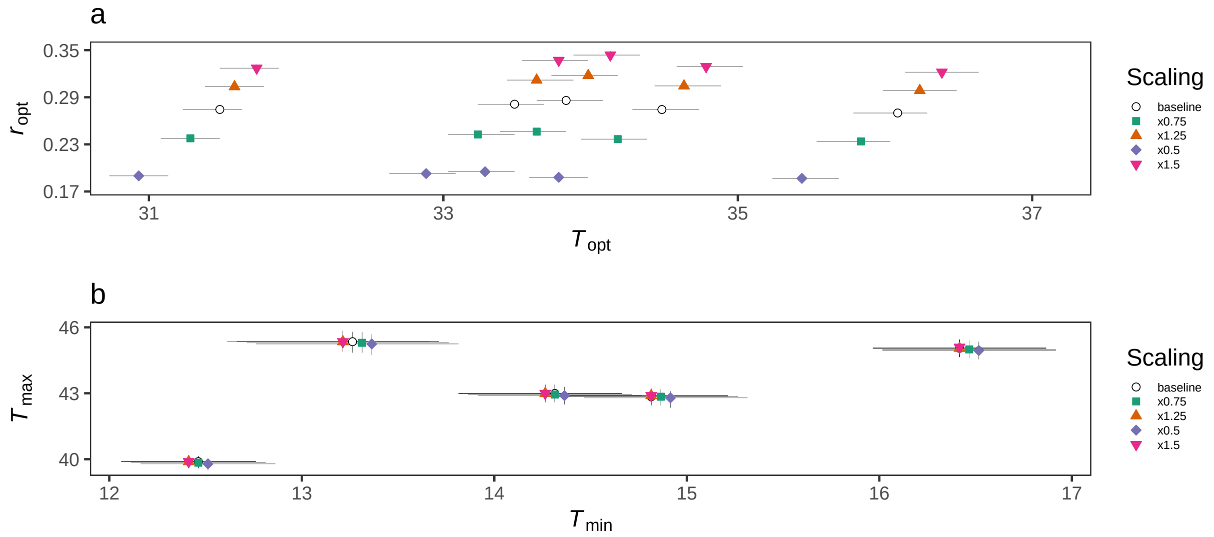

Results from uncontrolled evaporation experiments

Figure S4: Uncontrolled evaporation: Relative humidity shapes the temperature dependence of juvenile fitness traits in *An. stephensi* **(a–f)**. a. Humidity–dependent survival probability TPCs (p_{EA} in Eqn. 2, Main text). b–c. **b.** Numerical survival probability parameter estimates of T_{min} and T_{max} (Table S8). **c.** Predicted peak survival probabilities at T_{pk} at each humidity level (Table S9). Legend in b also applies to c. **d.** Humidity–dependent development rate TPCs ($1/\alpha$ in Eqn. 2). **e.** Development rate parameter estimates for T_{min} versus T_{max} across humidity levels (Table S11). **f.** Predicted peak development rate at T_{pk} at each humidity level (Table S10). Prediction bounds in **a** and **d** are HPD intervals estimated from the posteriors for each TPC. In b–c and e–f, error bars represent 95% credible intervals summarizing posterior uncertainty.

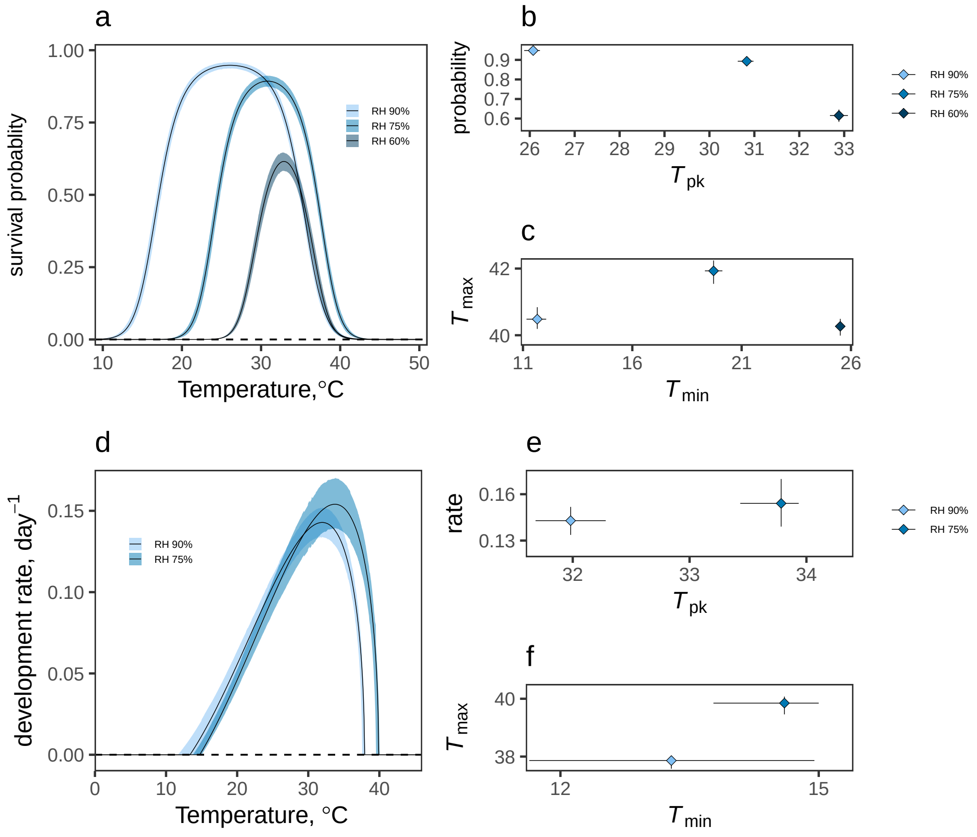

Figure S5: Uncontrolled evaporation: Effects of relative air humidity on juvenile fitness traits shape the temperature dependence of maximal population growth rate, r_m. (a–c) **a.** r_m TPCs across relative humidity levels. (b–c) **b.** r_{opt}s versus T_{opt}s (Table S12), **c.** T_{min} versus T_{max} across humidity levels (Table S13). Prediction bounds in **a** are HPD intervals calculated using the posteriors for each humidity–dependent TPC. Error bars in **b** and **c** represent 95% credible intervals summarizing posterior uncertainty.

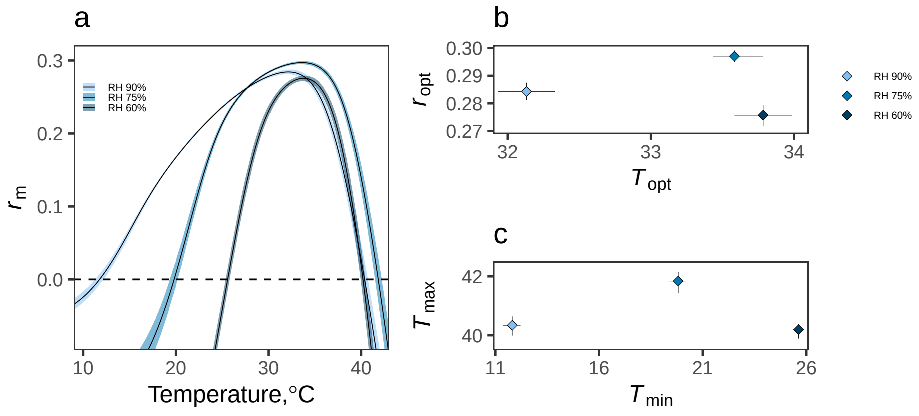

Figure S6: Uncontrolled evaporation: Relative air humidity and temperature interact to modulate the temperature–size relationship in ***Anopheles stephensi*****.** Regression lines show that body size decreased with temperature (Slope: –0.10998; CrI: –0.1397837, –0.0805072) and humidity (Slope: –0.0301336; CrI: –0.0412171, –0.0191704), and body size decreased more steeply with temperature at lower humidity (Interaction: 0.0007560; CrI: 0.0004171, 0.0010998). Boxplot horizontal lines represent medians; lower and upper hinges are the 25th and 75th percentiles. Upper whiskers extend from the hinge to the largest value no further than 1.5 × inter-quartile range (IQR) from the hinge. The lower whisker extends from the hinge to the smallest value at most 1.5 × IQR of the hinge. Points represent individual mosquitoes.

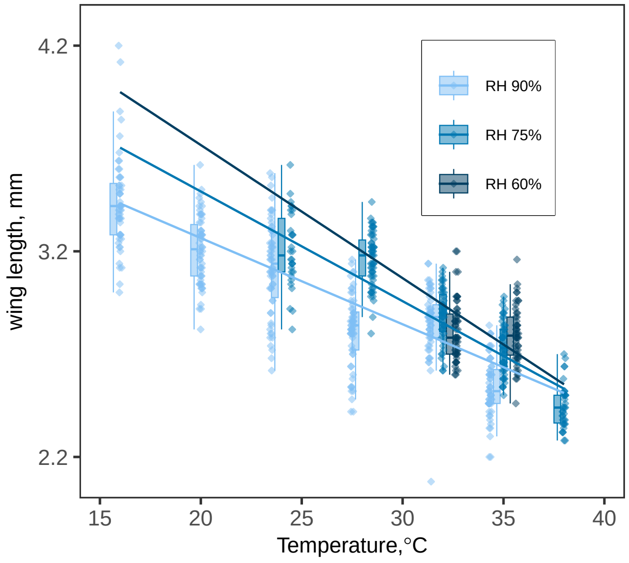

Table S8: Estimates for the juvenile survival TPC parameters; B_{pk} and T_{pk}. Numerical posterior estimates (median ± 95% credible intervals) of the parameters from n = 10,000 iterations for a burn–in of n = 5000.

RH (%)	Bpk median	Bpk lower	Bpk upper	Tpk median	Tpk lower	Tpk upper
90	0.948	0.937	0.958	26.076	25.876	26.226
75	0.893	0.873	0.912	30.831	30.631	30.981
60	0.615	0.584	0.647	32.883	32.683	33.083

Table S9: Estimates for the juvenile survival TPC parameters; T_{min} and T_{max}. Numerical posterior estimates (median ± 95% credible intervals) of the parameters from n = 10,000 iterations for a burn–in of n = 5000.

RH (%)	Tmin	Tmin lower	Tmin upper	Tmax	Tmax lower	Tmax upper
90	11.662	11.161	12.062	40.490	40.190	40.841
75	19.720	19.219	20.070	41.942	41.542	42.242
60	25.526	25.275	25.676	40.290	39.990	40.490

Table S10: Estimates for the juvenile development rate TPC parameters; B_{pk} and T_{pk}. Numerical posterior estimates (median ± 95% credible intervals) of the parameters from n = 10,000 iterations for a burn–in of n = 5000.

RH (%)	Bpk median	Bpk lower	Bpk upper	Tpk median	Tpk lower	Tpk upper
90	0.140	0.131	0.150	31.982	31.582	32.282
75	0.153	0.138	0.169	33.784	33.433	33.984

Table S11: Estimates for the juvenile development rate TPC parameters; T_{min} and T_{max}. Posterior estimates (mean ± 95% credible intervals) of the standard Briere model (SE2) parameters from n = 10,000 iterations for a burn–in of n = 5000.

RH (%)	Tmin	Tmin lower	Tmin upper	Tmax	Tmax lower	Tmax upper
90	13.238	11.442	14.994	37.836	37.469	38.016
75	14.602	13.714	15.000	39.839	39.444	40.084

Table S12: Estimates for the r_m TPC parameters; T_{opt} and r_{opt}. Numerical posterior estimates (median ± 95% credible intervals) of the parameters from n = 10,000 iterations for a burn–in of n = 5000.

RH (%)	ropt median	ropt lower	ropt upper	Topt	Topt lower	Topt upper
90	0.284	0.281	0.287	32.132	31.932	32.332
75	0.297	0.295	0.299	33.584	33.433	33.784
60	0.276	0.272	0.280	33.784	33.584	33.984

Table S13: Estimates for the r_m TPC parameters; T_{min} and T_{max}. Numerical posterior estimates (median ± 95% credible intervals) of the parameters from n = 10,000 iterations for a burn–in of n = 5000.

RH (%)	Tmin	Tmin lower	Tmin upper	Tmax	Tmax lower	Tmax upper
90	11.812	11.361	12.212	40.340	40.040	40.641
75	19.820	19.419	20.220	41.842	41.441	42.142
60	25.626	25.375	25.776	40.190	39.890	40.390

Figure S7: Uncontrolled evaporation: Observed juvenile survival data (hatching–to–adult) across temperature–humidity levels. There were n=3 replicates per treatment each containing n=100 L1 larvae at the start of the experiment. The figure shows the number of survivors for each treatment from a pooled total of n=300 at the start of the experiment. No individuals survived to adulthood at 14°C and 42°C irrespective of humidity level.

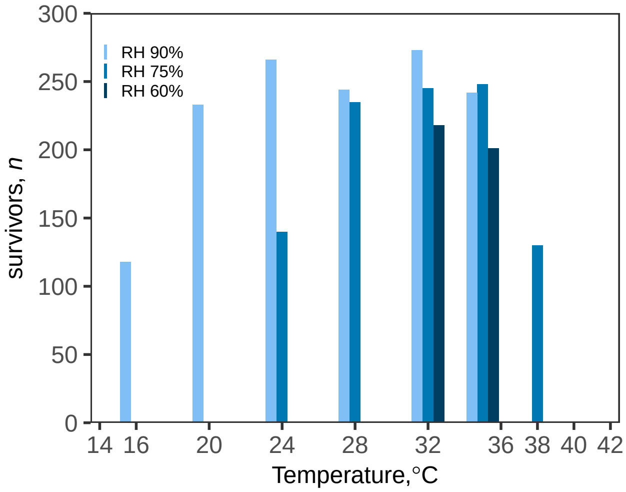

Figure S8: Uncontrolled evaporation: Juvenile development time TPCs (hatching–to–adult) used for the temperature– and humidity–dependent r_{m} TPCs. Development time (α in Eqn. 2) TPCs were fitted using Eqn. SE1. Points are individual mosquitoes.

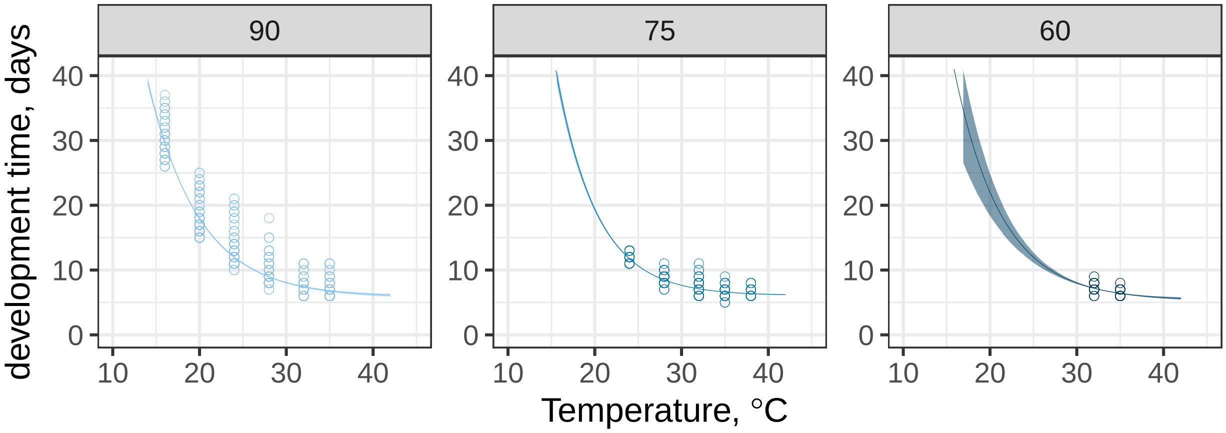

Figure S9: Seasonal mean temperatures and relative humidity across Africa and South Asia under historical climate conditions (1970–2000). Panels (a) and (c) show seasonal mean temperatures across Africa and India for 1970–2000, respectively. Panels (b) and (d) show mean annual relative humidity by season across Africa and India for the same period, respectively.

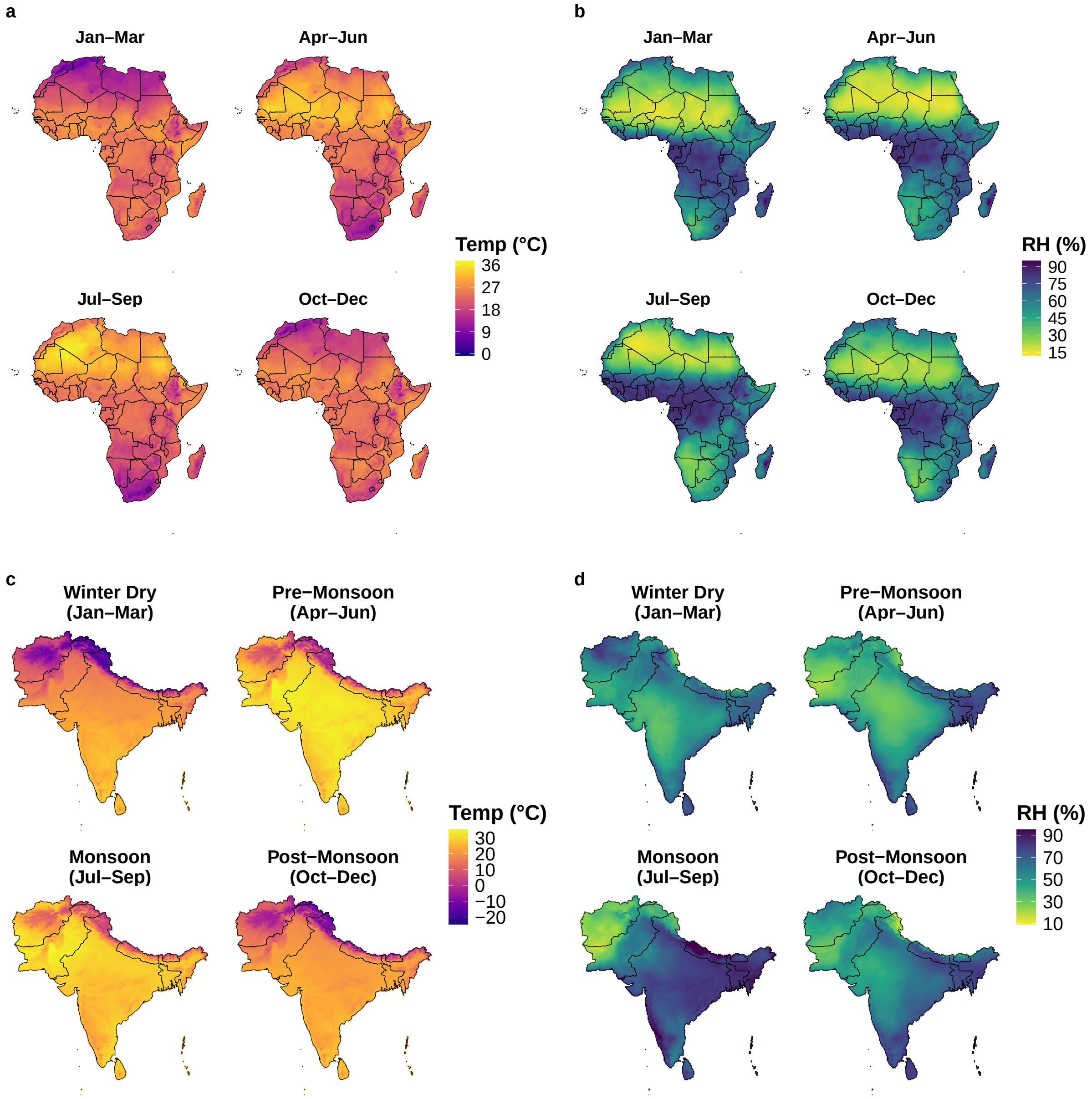

Literature Cited

Atkinson, D. (1995). Effects of temperature on the size of aquatic ectotherms: Exceptions to the general rule. *Journal of Thermal Biology*, 20(1-2):61–74.

Briegel, H. (1990a). Fecundity, metabolism, and body size in *Anopheles* (Diptera: Culicidae), vectors of malaria. *Journal of Medical Entomology*, 27(5):839–850.

Briegel, H. (1990b). Metabolic relationship between female body size, reserves, and fecundity of *Aedes aegypti*. *Journal of insect physiology*, 36(3):165–172.

Briere, J.-F., Pracros, P., Le Roux, A.-Y., and Pierre, J.-S. (1999). A novel rate model of temperature-dependent development for arthropods. *Environmental Entomology*, 28(1):22–29.

Bürkner, P.-C. (2017). `brms`: An `R` package for Bayesian multilevel models using Stan. *Journal of Statistical Software*, 80(1):1–28.

Cator, L. J., Johnson, L. R., Mordecai, E. A., El Moustaid, F., Smallwood, T. R., LaDeau, S. L., Johansson, M. A., Hudson, P. J., Boots, M., Thomas, M. B., et al. (2020). The role of vector trait variation in vector-borne disease dynamics. *Front. Ecol. Evol.*, 8:189.

Miazgowicz, K., Shocket, M., Ryan, S. J., Villena, O., Hall, R., Owen, J., Adanlawo, T., Balaji, K., Johnson, L. R., Mordecai, E. A., et al. (2020). Age influences the thermal suitability of *Plasmodium falciparum* transmission in the Asian malaria vector *Anopheles stephensi*. *Proceedings of the Royal Society B*, 287(1931):20201093.

Pathak, A. K., Shiau, J. C., Thomas, M. B., and Murdock, C. C. (2019). Field relevant variation in ambient temperature modifies density-dependent establishment of *Plasmodium falciparum* gametocytes in mosquitoes. *Frontiers in Microbiology*, 10:2651.

R Core Team (2023). *R: A Language and Environment for Statistical Computing*. R Foundation for Statistical Computing, Vienna, Austria.

Sorek, S., Smith Jr., J. W., Huxley, P. J., and Johnson, L. R. (2025). `bayesTPC`: Bayesian inference for thermal performance curves in `R`. *Methods in Ecology and Evolution*.
